# Relationship Between BMI, Self-Rated Depression, and Food Addiction—A Cross-Sectional Study of Adults in Postpandemic Poland

**DOI:** 10.1155/2024/5563257

**Published:** 2024-10-28

**Authors:** Magdalena Zielińska, Edyta Łuszczki, Anna Bartosiewicz, Łukasz Oleksy, Artur Stolarczyk, Katarzyna Dereń

**Affiliations:** ^1^Institute of Health Sciences, College of Medical Sciences of the University of Rzeszow, Rzeszów 35-310, Poland; ^2^Faculty of Health Sciences, Department of Physiotherapy, Jagiellonian University Medical College Krakow, Krakow 31-008, Poland; ^3^Department of Orthopaedics, Traumatology and Hand Surgery, Faculty of Medicine, Wroclaw Medical University, Wroclaw 50-556, Poland; ^4^Orthopedic and Rehabilitation Department, Medical Faculty, Medical University of Warsaw, Warsaw 02-091, Poland

**Keywords:** BMI, depression, eating behavior, mental health, obesity

## Abstract

Depression and obesity are two diseases that have a profound impact on global health. The relationship between obesity and depression is strongly comorbid, tending to exacerbate metabolic and depressive symptoms. Research also shows that there are complex interactions between obesity, depression symptoms, and food addiction (FA). The aim of the study was to investigate the relationship between body mass index (BMI), self-rated depression, and FA. The study sample consisted of 735 subjects (90.2% female, *M*_BMI_ = 27.58 kg/m^2^, standard deviation (SD)_BMI_ = 6.58 kg/m^2^) aged 18–70 years (*M* = 39.01, SD = 14.07). The prevalence of FA symptoms was measured using the Yale Food Addiction Scale 2.0 and self-rated depression was assessed using the Zung Self-Rating Depression Scale. The prevalence of FA in the group was 41% and self-rated depression was present in 34.1% of the participants. It was found that the higher the BMI of the subjects, the higher the severity of FA, but the BMI was not significantly correlated with the severity of depression. In addition, the greater the severity of self-rated depression, the greater the severity of FA. This correlation was stronger for men than for women in the 18–30 and 31–45 age groups. The severity of self-rated depression and FA was significantly higher in people with an eating disorders. This study adds to the growing body of evidence suggesting that the presence and severity of FA are associated with the severity of self-rated depression, particularly in younger adults. In addition, people with a high BMI, indicating obesity, are more likely to have FA, especially severe FA.

## 1. Introduction

For many years, there has been an ongoing debate in the scientific community about the addictive potential of highly processed foods and addictive eating behaviors in the context of overconsumption and obesity, and the term “food addiction” (FA) is becoming increasingly common in modern society [1, 2]. There is still no consensus on the clinical definition of FA [3]. However, there has been a recent resurgence of interest in the subject among researchers [4]. FA is a condition characterized by a lack of control over food intake, excessive food consumption despite negative health or social consequences, and repeated unsuccessful attempts to control intake [5]. Current scientific analyses focus on better understanding the factors, mechanisms, and behaviors associated with overeating and verifying whether disordered food intake, particularly in the context of obesity, fits the addiction model [6, 7]. FA can be associated with a variety of health problems, ranging from psychological and/or psychiatric (e.g., low self-esteem, depressed mood, major depressive disorder [MDD], binge eating disorder [BED]) to somatic (e.g., excess body weight, metabolic imbalances) or social (e.g., social isolation) [8]. To date, some studies suggest neurobiological similarities between substance dependence and FA, such as changes in the reward system, neurotransmitters, and impulse control systems [9]. Furthermore, people with obesity and substance use disorders (SUDs) have a reduced number of dopamine D2 receptors in the brain [10]. People with obesity have been shown to have reduced expression of dopamine receptors in the brain, especially in severe obesity, which may indicate reduced sensitivity to food-related reward stimuli [11]. According to the researchers, ultra-processed foods (UPFs), which are high in both added fats and refined carbohydrates, are currently the main contributors to FA [12, 13]. Moreover, under the influence of negative emotions, people often reach for foods rich in these macronutrients [14]. In fact, FA is a very complex construct, covering the clinical components of eating disorders (i.e., lack of control over eating behavior, especially in relation to palatable foods) and SUDs (e.g., impulsive personality traits) and obsessive–compulsive disorder (i.e., intrusive thoughts related to food stimuli) [15]. To date, FA has not been officially included as a separate category in the Diagnostic and Statistical Manual of Mental Disorders, Fifth Edition (DSM-5) or the International Classification of Diseases, 11th Revision (ICD-11), but is based on the criteria for addictive disorders set out in these diagnostic manuals [16, 17].

Depression and obesity are two diseases that have a profound impact on global health [18]. With the co-occurrence of obesity and depression and the growing interest in FA, it is urgent to verify and understand the relationship between these phenomena and FA [19]. To date, a higher prevalence of FA has been shown in people with a higher body mass index (BMI) [20]. The relationship between obesity and depression is strongly comorbid, tending to exacerbate metabolic and depressive symptoms [21]. Excess body weight can also lead to feelings of loneliness or social isolation, for example, due to stigma, increasing the risk of psychiatric disorders [22]. Research also shows that there are complex interactions between FA and the severity of depression [23]. An increased consumption of palatable foods is often observed during periods of emotional distress [24]. Neurocognitive dysfunction associated with depression shows impairments in reward processing, which can increase the probability of addictive eating behaviors [25]. The aim of the study was to investigate the relationship between BMI, self-rated depression, and FA. Therefore, based on previous research, the following hypotheses were formulated:

1. Selected sociodemographic factors will significantly discriminate the severity of self-rated depression and the severity of FA in study adults.
2. There will be a significant correlation between age and sex and the severity of self-rated depression and the severity of FA in the study adults.
3. There is a significant correlation between BMI and the severity of self-rated depression and the severity of FA in the study adults.
4. The severity of self-rated depression and the severity of FA are significantly higher among those who reported the presence of comorbidities, especially eating disorders.

## 2. Materials and Methods

### 2.1. Participants

This cross-sectional study was conducted in the second half of 2022, after the pandemic status of COVID-19 had been lifted in Poland. A total of 750 people participated in the study. The sampling method was nonprobabilistic, through invitations to participate in the study through a social networking site (Facebook). A graphic invitation to the survey and a link to the forms, preceded by a description of the survey, were shared on social networks (various online forums, Facebook groups). Participants were able to share the information with a link to the survey on social media (snowball sampling). After accessing the link, the participants received access to information about the study. They gave their informed consent to participate before beginning to complete the questionnaires. Participants could complete the questionnaire directly from their smartphone, tablet, or computer. They could withdraw from the study at any time. In the end, 735 participants who met the inclusion criteria were eligible for the study. Fifteen participants were excluded because they did not meet the study criteria. These included people under the age of 18 years of age and pregnant and breastfeeding women. A total of 735 subjects (663 women, 72 men) aged 18–70 years (*M* = 39.01, SD = 14.07) participated in the study. The subjects' BMI ranged from 16.1 to 53.8 kg/m^2^ (*M* = 27.58 kg/m^2^, SD = 6.58).

### 2.2. Measures

All participants completed online questionnaires in Polish that included information on sex, age, weight, height, education, work situation, place of residence, and presence of comorbidities, including eating disorders. The participants then completed two questionnaires: The Yale Food Addiction Scale 2.0 (YFAS 2.0) and the Zung Self-Rating Depression Scale (SDS), which were designed to assess FA and self-rated depression, respectively. The internal consistency of the questionnaires was checked using the Cronbach's *α* coefficient to minimize response bias. Body weight and height were taken as reported by the respondents. BMI was calculated using the formula: body weight/height (m^2^). World Health Organization standards were used to classify participants into BMI categories: underweight (BMI < 18.5 kg/m^2^), normal weight (BMI 18.5–24.99 kg/m^2^), overweight (BMI 25.00–29.99 kg/m^2^), obesity class I (BMI 30.00–34.99 kg/m^2^), obesity class II (BMI 35.00–39.99 kg/m^2^), and obesity class III (BMI ≥ 40.00 kg/m^2^) [26].

### 2.3. Psychometric Measurements

#### 2.3.1. YFAS 2.0

The YFAS 2.0 is a self-report questionnaire, updated in 2016, which has been validated for the evaluation of food addictive behavior [27–29]. Based on this, the Polish adaptation questionnaire identified the symptoms of FA in the study group [30, 31]. In YFAS 2.0, all 35 questions are scaled from 0 to 7 (from *never* to *daily*) and ask participants to think about specific foods, such as highly processed foods. Based on the 11 diagnostic criteria for SUD in DSM-5 (e.g., craving, tolerance, or withdrawal), a symptom score of 0–11 is calculated, reflecting the severity of FA symptoms. In addition, clinically significant impairment or distress due to eating behavior is evaluated. The presence of no more than one symptom or the absence of symptom 12 (substance use causes serious problems or distress) was classified as nondependence. FA is diagnosed when two or more symptoms are present together with symptom 12 (as mild dependence), 4–5 symptoms together with symptom 12 were classified as moderate dependence, and the presence of more symptoms together with 12 was classified as severe dependence. FA is not listed as a substance-related and addictive disorder in DSM-5, so the presence of symptoms and the degree of FA cannot be considered from a clinical point of view, but only from a scientific point of view. It simply means meeting the YFAS 2.0 criteria for FA. The Cronbach's *α* value was found to be 0.86 [31]. In this study, the Cronbach's value for the whole scale was set at 0.82. The authors have permission to use this instrument from the copyright holders.

#### 2.3.2. Zung SDS

The Zung SDS is a tool to assess the severity of depressive symptoms in adults and is well suited for screening purposes [32]. This short scale is widely used in all age groups for both screening and measuring depression, particularly in research contexts [33, 34]. It includes 20 symptoms divided into four groups: mood disturbances, physiological functions, psychomotor activity, and psychiatric symptoms. The scale uses affirmative sentences (10 describes pathological phenomena, 10 refers to the presence of normal features). The respondent's task was to indicate how often he or she observed a particular characteristic in himself or herself. Each negative statement is scored on a scale of 1–4 points, while each positive statement is scored on a scale of 4–1 points. The total SDS score can range from 20 to 80 points, with higher scores indicating a greater severity of depressive symptoms [31]. The number of points scored indicates <50 points: (1) no depressive symptoms, 50–59 points; (2) those with mild to moderate depression, 60–69 points; (3) those with moderate to severe depression, ≥70 pts; and (4) those with severe to very severe depression [35]. For the 50-point cutoff applied by many researchers, sensitivity is 78.9% and specificity is 83.7% [36]. The variability of the cutoff values used in the literature is used to maximize the benefit of testing. A cutoff point ≥50 is recommended [37]. SDS has also been validated to identify clinically significant depression symptoms in older people compared to the Beck Depression Inventory, 21-item version (BDI-21), which is one of the most widely used screening scales for depression [38]. The SDS is relatively easy to use and easy to complete, making it a popular tool in both research and clinical practice. The internal consistency of the SDS is high (Cronbach's *α* 0.81) [39]. The present study showed high internal consistency (Cronbach's *α* = 0.86). Furthermore, a negative correlation was observed between the total score and the zung7 item (*I notice that I am losing weight*).

### 2.4. Ethics

The study was carried out according to the Declaration of Helsinki and was approved by the Bioethics Committee of the University of Rzeszów (Resolution No. 2022/073 of June 15, 2002). All participants were informed of the aims and objectives of the study and all gave their informed consent to take part in the study.

### 2.5. Data and Statistical Analysis

Calculations were carried out in the statistical environment R ver.3.6.0, in Predictive Software Project (PSPP) software and MS Office 2019. *p*=0.05 was used as the level of statistical significance. Variables expressed at the ordinal or nominal level were analyzed using tests based on the chi-square distribution. For 2 × 2 tables, a continuity correction was applied, while for tables larger than 2 × 2, when the conditions for using the chi-square test were not met, Fisher's exact test with extension was used. Nonparametric tests (Mann–Whitney *U* test or Kruskal–Wallis test) were used to analyze quantitative variables presented by the groups. The Spearman's rank correlation coefficient was used to test the association between variables. The tests were selected based on the distribution of the variables, which was verified with the Shapiro–Wilk test.

## 3. Results

The major patient demographic and other characteristics include physiological factors, comorbidities, sex, age and age groups, education level, place of living, employment status, comorbidities, eating disorders, nutritional status (underweight, normal body weight, overweight and classes I, II, and II obesity), self-rated depression, and symptoms of FA and other descriptive characteristics of the study subjects are shown in Table 1.

Descriptive statistics, including mean, minimum, maximum, and median values of variables such as age, weight, height, calculated BMI, and results of standardized questionnaires, are presented in Table 2 for the study group *N* = 735 subjects.

### 3.1. Sociodemographic Factors Versus FA and Severity of Self-Rated Depression

Occupational activity did not make a statistically significant difference between the severity of depression and FA. Due to the extremely small numbers in the *primary*, *lower secondary*, and *vocational education* categories, they were combined with the *secondary education* category. Education was also shown to not significantly differentiate the severity of depression and FA. The results of the Mann–Whitney *U* test for independent samples are shown in Table 3.

The place of residence also did not make a statistically significant difference between the severity of depression and FA. The results of the Kruskal–Wallis test are shown in Table 4.

### 3.2. Correlation Between the Severity of Self-Rated Depression and FA According to Age and Sex

**Figure 1:**
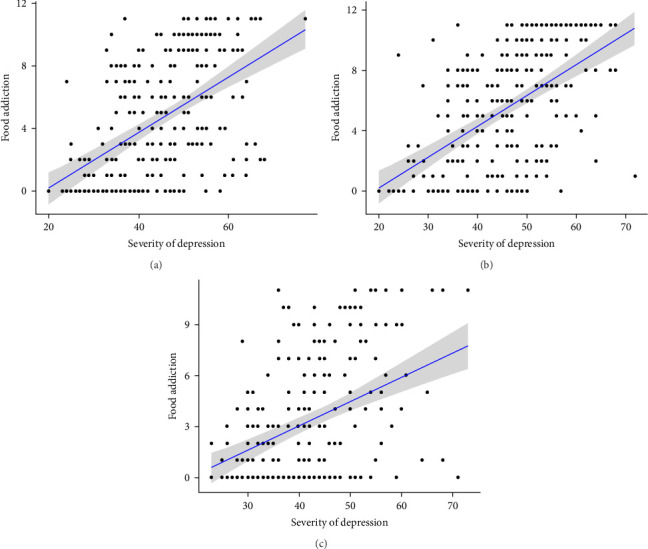
Severity of depressive symptoms and FA in relation in (a) 18–30 year group, (b) 31–45 year group, and (c) 46–70 year group. FA, food addiction.

There was a statistically significant correlation between age and the severity of self-rated depression (*ρ* = −0.125, *p*=0.001) and FA (*ρ* = −0.158, *p* < 0.001). These were weak and negative correlations, which means that the older the respondent, the lower the severity of depression and FA. In addition, the correlation between these aspects was checked in the different age groups. There was a statistically significant positive correlation between depression severity and FA in the 18–30 and 31–45 age groups (*p* < 0.001). This correlation was strong, with coefficients of *ρ* = 0.528 and *ρ* = 0.552, respectively. The greater the severity of depression, the greater the severity of FA. The 46–70 age group also showed a statistically significant (*p* < 0.001), moderately strong (*ρ* = 0.372), and positive correlation between the severity of depression and FA. The strength of the correlation was shown to be very similar in the 18–30 and 31–45 age groups, but much weaker in the 46–70 age group. The described correlations are shown in Figure 1.

In addition, the above correlations in the age groups were also examined by sex. There was a statistically significant positive correlation between FA and self-rated depression severity in all age groups by sex (except for men in the 46–70 age group). This means that higher FA scores were associated with a higher severity of depression. The correlation was stronger for men than for women in the 18–30 and 31–45 age groups. In the age group 46–70, the correlation was significant for women but not for men. For both sexes, the relationship between FA and self-rated severity of depression appeared to be strongest in younger age groups and weaker in older age groups. The results are presented in Table 5.

### 3.3. Correlation Between BMI and Severity of Self-Rated Depression and Severity of FA

**Figure 2:**
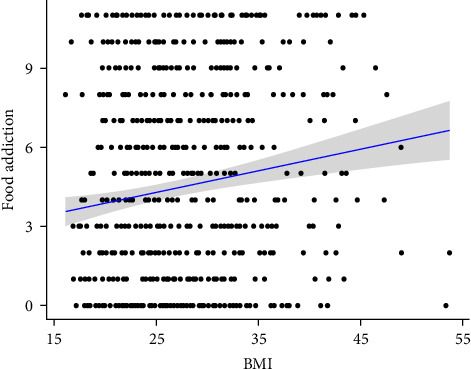
Correlation between BMI and severity of FA. BMI, body mass index; FA, food addiction.

There was a statistically significant correlation between BMI and FA (*p* < 0.001). The correlation was weak (*ρ* = 0.135), but positive, which means that the higher the BMI, the greater the severity of FA. The distribution of the variables is illustrated in Figure 2.

However, there was no statistically significant correlation between BMI and depression severity (*p*=0.295, *ρ* = 0.039).

**Figure 3:**
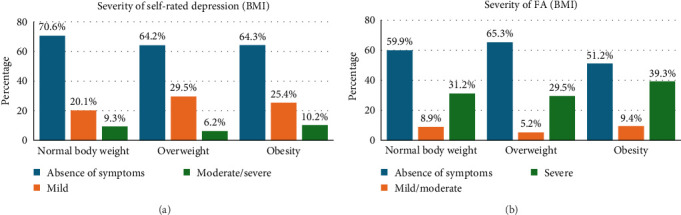
Prevalence of depressive symptoms (a) and severity of FA (b) in relation to BMI. BMI, body mass index; FA, food addiction.

Furthermore, for the purposes of the study, the BMI category *underweight* was excluded and the category class I *obesity* was combined with *class II obesity* and *class III obesity*. The categories of *severe* to *moderate* depression and *mild* to *moderate* FA were also combined. The Chi-square test was used to verify the existence of an association. For the severity of self-rated depression, the result was not statistically significant (*p*=0.119), indicating that there were small differences in the distribution of the severity of self-rated depression by BMI (*χ*2 = 7.349, *df* = 4). This showed that depression scores were not significantly dependent on BMI. The study result was statistically significant for FA severity (*p*=0.034). This indicates the presence of large differences in the distribution of FA severity by BMI category (*χ*2 = 10.407, *df* = 4). FA was shown to be statistically significantly more common in subjects with overweight than in other subjects, and severe FA was significantly more common in people with obesity. The data are presented in Figure 3.

In summary, a significant relationship was observed between BMI and the severity of FA. No significant relationship was found between BMI and self-rated depression.

### 3.4. Comorbidities and Severity of Self-Rated Depression and FA Among Surveyed Adults

For the purposes of the study, all categories of present comorbidities were combined, resulting in two response categories: *present* and *no comorbidities* (*absent*). The severity of self-rated depression was shown to be statistically significantly higher in those with comorbidities than in those without comorbidities. The presence of comorbidities did not significantly differentiate the severity of FA. The severity of depression and FA was also shown to be statistically significantly higher among those with eating disorders. The relationships described are shown in Table 6.

## 4. Discussion

The main objective of the present study was to determine the prevalence of FA in Polish adults after the pandemic after COVID-19 and to identify possible differences in prevalence, mainly with respect to BMI, comorbidities, and the association of FA with self-rated depression. To our knowledge, the present study is the first in our country to examine the association between FA and self-rated depression and the variables mentioned above.

Studies on the prevalence of FA based on YFAS have shown that the prevalence among adults ranges from 5% to 20% in the general population [40]. It is particularly high among people with excess body weight, especially with obesity (25%–32%) and eating disorders (70%–96%) [41–45]. In this study, 41% of the group met the diagnosis of FA symptoms based on YFAS 2.0. The prevalence of FA in the present study was found to be much higher than the results of similar studies in the literature and much higher (almost three times) than a study assessing the prevalence of FA during the COVID-19 pandemic in Poland (14.1%) [46]. The researchers found similar results in a recently published study of university students in Turkey. In this group, 40% were affected by FA [47]. In a recent survey of students in Spain, 31.9% of the participants met the criteria for FA [48]. A possible reason may be the inclusion of people with eating disorders in the current study, compared to work at the time of COVID-19 in Poland, where these people were excluded [46]. Furthermore, 59.5% of the respondents in our study were people with overweight or obesity and, as mentioned above, the prevalence of FA is higher in these groups. Additionally, it is likely that the difference may be due to the timing of response collection—this was after the pandemic. Changes caused by the pandemic period, that is, high level of stress, social isolation, may have influenced the higher prevalence of FA and also the high prevalence of self-rated depression (34.1%) in this group [49, 50]. For symptoms of FA according to YFAS, 3.7% of the respondents had mild FA, 4.4% of the group had moderate FA, and 32.9% of the respondents had symptoms of severe FA. Similarly, in Burrows et al.'s [51] study, although at a much lower level, severe FA affected the largest proportion of the group (18.9%), moderate (2.6%) and mild (0.6%). In terms of depressive symptoms, a 2019 survey of a random sample of 2413 people in Poland found depressive symptoms in 23.4% of men and 33.4% of women [52]. A study by Brągiel and Gambin [53] of depressive symptoms in 110 Polish adults in the context of both the war in Ukraine and the COVID-19 pandemic found that 52.7% of participants reported subjective severity of depressive symptoms. It should be noted that most people with depressive symptoms in our study had mild to moderate depression symptoms (25.4%), moderate to major depression was reported by 8.2%, and severe to very severe by only 0.5%. Importantly, although the subjects were recruited through publicly available social media invitations, their results show a high prevalence of FA and depressive symptoms, suggesting that they may be actively seeking help and treatment.

1. *In the present study, work activity, place of residence, and education did not differ significantly between the severity of self-rated depression and FA.*
  The level of education and the place of residence can affect access to health care and health outcomes [54, 55]. The protective effect of educational attainment on depression varies between subgroups of the population. Throughout life, the protective effect of educational attainment on depression appears to be curvilinear, with greater effects among young adults and older adults, but this remains an active area of research due to conflicting findings [56]. In the study by Piwoński et al. [52], depressive symptoms were more common in older people and those with primary education. Other factors, such as work hours, work conditions, work intensity, high demands, and passive work, are relevant to the relationship between depressive symptoms and work [57–59]. The prevalence of depressive symptoms was higher among urban than rural residents, although the study results are not conclusive on this point [60, 61]. In the case of FA and education, Ayaz et al. [62] showed that as education levels increased, symptoms of FA decreased significantly. Interestingly, a recent study showed in the results of mediation analysis that the relationship between workaholism and depression, anxiety, and stress is mediated by FA [63]. Our findings are consistent with previous studies, which also failed to confirm an association between FA and severity of depression and educational level, work activity, and place of residence, suggesting that other factors may play a more important role in the development of these disorders in the development of these disorders [59, 61]. In future research, it would be worthwhile to extend the work activity of the respondents to include issues of working time, work intensity, and work conditions in relation to FA.
2. *The higher the severity of self-rated depression, the higher the severity of FA, particularly in the age groups of 18–30 and 31–45. The older the subjects, the significantly lower the severity of self-rated depression and FA.*
  Clinical trials have reported a strong association between depressive symptoms and the severity of FA, both in patients seeking weight loss and in those undergoing treatment, as well as in patients with eating disorders or type 2 diabetes [64, 65]. However, people with FA had scores that corresponded to a moderate severity of depressive symptoms. In the studies by Pape et al. [66] and Wiedemann et al. [67], the severity of FA symptoms was also positively correlated with the severity of depressive symptoms. Şanlier, Türközü, and Toka [69] also showed a positive association between FA and depression scores in 793 students, and Wattick et al. [68] showed that depression was the only significant predictor of FA in students. The results of the study by Meule et al. [70] showed that people with symptoms of FA reported more frequent hunger pangs, greater psychopathology of eating disorders, and more depressive symptoms. The results of our study are in line with the findings of the studies mentioned above. With regard to FA, most studies to date also suggest that in the nonclinical population, FA is more common in young adults. The results of recent systematic reviews and meta-analyses show a high prevalence of depressive symptoms in groups of older people [71, 72]. In the present study, the severity of depressive symptoms and FA was significantly lower with the age of the subjects. Furthermore, men in the 18–30 and 31–45 age groups showed a stronger correlation than women in the same age groups. In the age group 46–70, the correlation was significant for women but not for men. For both sexes, the relationship between FA and self-rated severity of depression appeared to be strongest in younger age groups and weaker in older age groups.
  Interestingly, in a recent study by Ceylan et al. [73], the relationship between FA and chronotype and mental stress was investigated. The risk factors for FA were female sex, evening chronotype, high BMI, and psychological pain. There was a strong direct correlation between chronotype and FA. In a cross-sectional study of 750 adults with overweight and obesity, Amicis et al. [74] showed that chronotype was associated with BED and FA in men, highlighting the link between chronobiology and sex differences as determinants of appetite and dysregulation of eating behavior and overweight and obesity. The results of previous studies also show that the chronotype tends to shift toward earlier hours of the day with age, and this trend is stronger in men than in women [75, 76]. In addition, the chronotype is believed to exert a substantial influence on mental health. In the case of depression, a late chronotype has been shown to be associated with an increased risk of depression in women, but not in men. The early chronotype is not associated with depression in women or men [77]. The association between circadian clock and its dysfunctions with metabolic derangements, depression, and cognitive dysfunctions even led some authors to redefine metabolic syndrome as ”circadian syndrome” [78]. Therefore, it is likely that age-related differences in depressive symptoms scores and FA can be explained, at least in part, by a reduction in the trend of the chronotype in the evening in older participants [76]. Unfortunately, any information on circadian clocks/chronotypes of study participants was left off-scope in this study. All related variables (BMI, depression scores, and FA) are closely coupled with the circadian clock/chronotype, which indicates further interesting research directions in this matter [79–82]. It is also important to remember that these results may be related to other factors, such as genetics, living environment, or life experiences, which may differ according to age and gender.
3. *It has been shown that the higher the BMI of the subjects, the greater the severity of FA, but the BMI did not correlate significantly with the severity of self-rated depression. Severe FA was significantly more common in people with obesity.*
  Many researchers highlight the prevalence of addictive eating behaviors, particularly in people with overweight and obesity [83–85]. Consistent with the findings of other researchers, this study also confirms the association between BMI and FA symptoms [86–88]. In a study by Hauck et al. [43], those who met the criteria for FA also had a higher BMI and were younger than those without FA. Güngör et al. [89] also showed in a case-control study in a group of patients with prebariatric surgery that the severity of FA was significantly associated with a higher BMI. In the study by Yousefi et al. [90], those with a class III obesity level had a significantly higher risk of developing FA than those with a class I obesity level. The results of the present study are in line with those of other researchers in relation to BMI and FA, but no significant relationship was found between BMI and the level of depression in the study group. Previous studies have shown that higher levels of depressive symptoms are associated with higher levels of BMI [91, 92]. Interestingly, in our study, the severity of depression decreased with age, while BMI tends to increase with age. Usta and Pehlivan [93], in a study of patients seeking obesity treatment, showed that depressive symptoms mediated the association between FA symptoms and BMI in people with obesity. A postpandemic study by Duan et al. [94] at Wuhan University in China also found that BMI was positively correlated with the severity of anxiety or depression. It is worth noting that the absence of an observed relationship between BMI and depression in our study may be due to differences in the research methodology, different characteristics of the study group, or other factors that can influence the results of the study that we did not consider, such as lifestyle [95]. Given the results obtained, it is worth considering whether FA is a factor in the psychological aetiology of obesity and whether the current failure of obesity treatment may be partly due to the lack of FA treatment [60]. In addition to being an important determinant of mental health, BMI is also an important determinant of general and reproductive health. However, there are growing concerns about the role of BMI as an ideal predictor and single indicator of general health, reproductive, and mental health outcomes, as it does not distinguish an important aspect, such as regional fat distribution [96–98].
4. *The severity of self-rated depression was significantly higher among those who reported comorbidities than among those without comorbidities, which was not shown in relation to FA. The severity of both FA and self-rated depression was significantly higher among those with eating disorders.*
  In this study, the highest percentages were observed in people with thyroid disease (21.9%), metabolic disease (17.41%), cardiovascular disease (17.28%), and eating disorders (9.12%). The severity of depression was significantly higher in those with comorbidities than in those without. In the study reported here, the presence of comorbidities did not significantly differentiate the severity of FA, but published data in the literature suggest that FA is strongly associated with the presence of type 2 diabetes [90, 99, 100]. The severity of depression and FA was significantly higher among those with an eating disorder, which is consistent with data in the literature [101]. Eating disorders can increase the risk of depression and FA because they can lead to serious health problems and affect mental functioning [102, 103]. Other comorbidities that do not necessarily directly affect eating behavior may be less relevant to the severity of FA, but may still contribute significantly to depression due to other factors such as pain or limitations in daily functioning [104, 105]. This suggests the need for more research on the links between FA and these conditions and potential mechanisms of co-occurrence. The worryingly high proportion of people in this study with excess body weight, depressive symptoms, and their co-occurrence with FA suggests that these health problems may have complex interrelationships and require multidisciplinary interventions [106].

### 4.1. Limitations of the Study

It should be emphasized that the results obtained must be interpreted in light of certain limitations. First, it is difficult to make causal claims based on this cross-sectional study. In addition, the use of social networks to recruit participants may lead to selection bias. The design of the online survey using self-reported data limits the generalisability of the results (may lead to an unrepresentative sample). Despite the increasing use and availability of the Internet in society, online questionnaires may be more accessible to certain groups (e.g., young, middle-aged people) than, for example, older people. Therefore, the prevalence of FA observed in the study sample described here should not be extrapolated to the prevalence of FA in the sample population. Some limitations also relate to potential errors and increased reliability due to self-reporting by study participants in the online survey, particularly self-reporting of height and weight. It should also be noted that this study did not distinguish between different types of eating disorders, such as anorexia, bulimia, or BED. More detailed analyses, taking into account the differences between these eating disorders, might lead to more precise conclusions. It should also be noted that the number of cases of these disorders in our study was small, suggesting that more research is needed in this area to obtain more representative results. The lack of consideration of the circadian preferences of the participants in our study is also a limitation. Given current reports on chronotype and the prevalence of depression and FA, it would be useful to include information on participants' biological clocks/chronotypes in future studies.

### 4.2. Strengths and Future Research Directions

One of the strengths of the present study is the relatively large sample of respondents, which increases the statistical power of the results. In addition to FA and self-rated depression, the study also considered other factors such as BMI, education, work situation, or the presence of comorbidities, including eating disorders, which can allow a more accurate interpretation of the results presented. Furthermore, the study design allowed us to examine FA in adults living in Poland after the pandemic, completed the prepandemic survey, and included aspects of mental health related to self-rated depression and the presence of eating disorders.

In future studies, it would be worthwhile to include lifestyle aspects such as smoking, alcohol consumption, stress levels, dietary behavior, and physical activity, and chronotype to further assess the complex relationships between body weight, FA, and mental status. Furthermore, our study, as most FA studies, showed a significant sex difference, with most participants being female, which requires further analysis and the inclusion of a larger group of men in studies of self-rated depression and FA.

Despite the lack of formal validation, the results of this work can be used as a basis for further research into the validation of the Zung scale in the Polish population, which may contribute to a better understanding of its precision and reliability in this context. The psychometric properties of this questionnaire used among people of different ages at the community level are needed to be assessed. A future research direction could be to include an eating style questionnaire in the work to help identify the complexity of the relationship between eating behavior and psychological (especially in the context of emotional eating and uncontrolled eating) and physiological parameters. More longitudinal studies are also needed to assess causal relationships between mental health and body weight.

## 5. Conclusions

This study adds to the growing body of evidence suggesting that the presence and severity of FA are associated with the severity of self-rated depression, particularly in younger adults. Interestingly, the correlation was stronger for men than for women in the 18–30 and 31–45 age groups. In the age group 46–70, the correlation was significant for women but not for men. In addition, people with a high BMI, indicating obesity, are more likely to have FA, especially severe FA. These results should be interpreted with caution and highlight the need for more longitudinal studies to assess the causal relationship between mental health and body weight.

A graphical summary of the study results is available in Figure S1.

## Figures and Tables

**Table 1:** Group characteristics.

Variable	Frequency	Percent
Sex		
Female	663	90.2
Male	72	9.8
Age range (years)		
18–30	253	34.4
31–45	253	34.4
46–70	229	31.2
Education level		
Primary	5	0.7
Lower secondary	5	0.7
Vocational	36	4.9
Secondary	265	36.1
Higher	424	57.7
Place of residence		
Village	184	25.0
City up to 250,000	272	37.0
City over 250,000	279	38.0
Professional activity		
Nonworking	189	25.7
Working	546	74.3
Comorbidities		
Metabolic diseases	128	17.41
Cardiovascular diseases	127	17.28
Thyroid diseases	161	21.9
Nervous system diseases	11	1.5
Respiratory system diseases	24	3.27
Gastrointestinal diseases	24	3.27
Eating disorders (BED, anorexia, bulimia, and others)	67	9.12
Cancer diseases	11	1.5
Osteoarticular diseases	6	0.82
Urinary diseases	1	0.14
Reproductive and other endocrine diseases	6	0.82
Not applicable	357	48.57
BMI classification		
Underweight	29	3.9
Normal body weight	269	36.6
Overweight	193	26.3
Obesity class I	152	20.7
Obesity class II	48	6.5
Obesity class III	44	6.0
Severity of self-rated depression		
Absence of symptoms	484	65.9
Mild	187	25.4
Moderate	60	8.2
Severe	4	0.5
Severbity of FA		
Absence of symptoms	434	59.0
Mild	27	3.7
Moderate	32	4.4
Severe	242	32.9

Abbreviations: BED, binge eating disorder; BMI, body mass index; FA, food addiction.

**Table 2:** Descriptive statistics of the study group.

Variable	*N*	*M*	*SD*	*Min*	*Maks*	*Me*
Age	735	39.01	14.07	18.00	70.00	37.00
Body weight	735	76.81	19.63	43.00	168.00	74.20
Body height	735	166.74	7.16	148.00	195.00	167.00
BMI index	735	27.58	6.58	16.10	53.80	26.50
Severity of self-rated depression	735	44.35	10.60	20.00	77.00	44.00
FA	735	4.49	3.86	0	11.00	4.00

Abbreviations: BMI, body mass index; FA, food addiction; *M*, mean; Maks, maximum; Me, median; Min, minimum; *N*, abundance; SD, standard deviation.

**Table 3:** Sociodemographic factors and FA and severity of self-rated depression.

Variable	*U*	*p*		Descriptive statistics	
			*Min*	*Maks*	*Me*
Professional activity					
Severity of depression	50,151.00	0.565	—	—	—
Nonworking	—	—	20.00	68.00	45.00
Working	—	—	20.00	77.00	44.00
FA	50,262.00	0.592	—	—	—
Nonworking	—	—	0	11.00	4.00
Working	—	—	0	11.00	4.00
Education level					
Severity of depression	63,486.50	0.390	—	—	—
Secondary or lower	—	—	20.00	73.00	45.00
Higher	—	—	20.00	77.00	44.00
FA	61,448.50	0.112	—	—	—
Secondary or lower	—	—	0	11.00	4.00
Higher	—	—	0	11.00	4.00

Abbreviations: FA, food addiction; Maks, maximum score; Me, median; Min, minimum; *p*, statistical significance; *U*, test statistics.

**Table 4:** Sociodemographic factors and FA and severity of self-rated depression.

Variable	Place of residence	*χ* ^2^	*df*	*p*	*Min*	*Maks*	*Me*
Severity of depression	Village	4.35	2	0.113	20.00	73.00	43.00
	City up to 250,000				20.00	72.00	45.00
	City over 250,000				23.00	77.00	45.00

FA	Village	5.35	2	0.069	0	11.00	4.00
	City up to 250,000				0	11.00	3.00
	City over 250,000				0	11.00	4.00

Abbreviations: *χ*^*2*^, test statistics; *df*, degrees of freedom; FA, food addiction; Maks, maximum score; Me, median; Min, minimum score; *p*, statistical significance.

**Table 5:** Correlations between severity of self-rated depression and FA by age and sex.

Age category (years)	Sex	Variable		FA	*p*-value for correlation
18–30	Female	Severity of depression	*rho*	0.520	*⁣* ^*∗∗∗*^
			*p*	<0.001	—
	Male		*rho*	0.719	*⁣* ^*∗∗∗*^
			*p*	<0.001	—
31–45	Female		*rho*	0.526	*⁣* ^*∗∗∗*^
			*p*	<0.001	—
	Male		*rho*	0.842	*⁣* ^*∗∗∗*^
			*p*	<0.001	—
46–70	Female		*rho*	0.365	*⁣* ^*∗∗∗*^
			*p*	<0.001	—
	Male		*rho*	0.346	—
			*p*	0.057	—

Abbreviations: FA, food addiction; *p*, significance; *rho*, Spearman's correlation coefficient.

*⁣* ^*∗*^ *p*  < 0.05; *⁣*^*∗∗*^*p*  < 0.01; *⁣*^*∗∗∗*^*p*  < 0.001.

**Table 6:** Presence of comorbidities versus severity of self-rated depression and FA.

	*U*	*p*	Descriptive statistics		
			*Min*	*Maks*	*Me*
Comorbidities					
Severity of depression	60,271.00	0.012	—	—	—
Present	—	—	23.00	73.00	45.00
Absent	—	—	20.00	77.00	44.00
FA	63,112.50	0.126	—	—	—
Present	—	—	0	11.00	4.00
Absent	—	—	0	11.00	3.00
Eating disorders					
Severity of depression	12,112.00	<0.001	—	—	—
Present	—	—	24.00	68.00	52.00
Absent	—	—	20.00	77.00	44.00
FA	10,322.50	<0.001	—	—	—
Present	—	—	0	11.00	9.00
Absent	—	—	0	11.00	3.00

Abbreviations: FA, food addiction; Maks, maximum score; Me, median; Min, minimum score; *p*, statistical significance; *U*, test statistics.

## Data Availability

The data presented in this study are available upon request from Magdalena Zielińska. The data are not publicly available.

## Supporting Information

Supporting Information A graphic summary of the conducted research, briefly describing the subjects, methods, most important results, and conclusions.
