# Supplementary figures and images for: Relationship Between BMI, Self-Rated Depression, and Food Addiction—A Cross-Sectional Study of Adults in Postpandemic Poland
Source: Depress Anxiety. 2024 Oct 28;2024:5563257. doi: 10.1155/2024/5563257 (PMC11921829; doi:10.1155/2024/5563257)

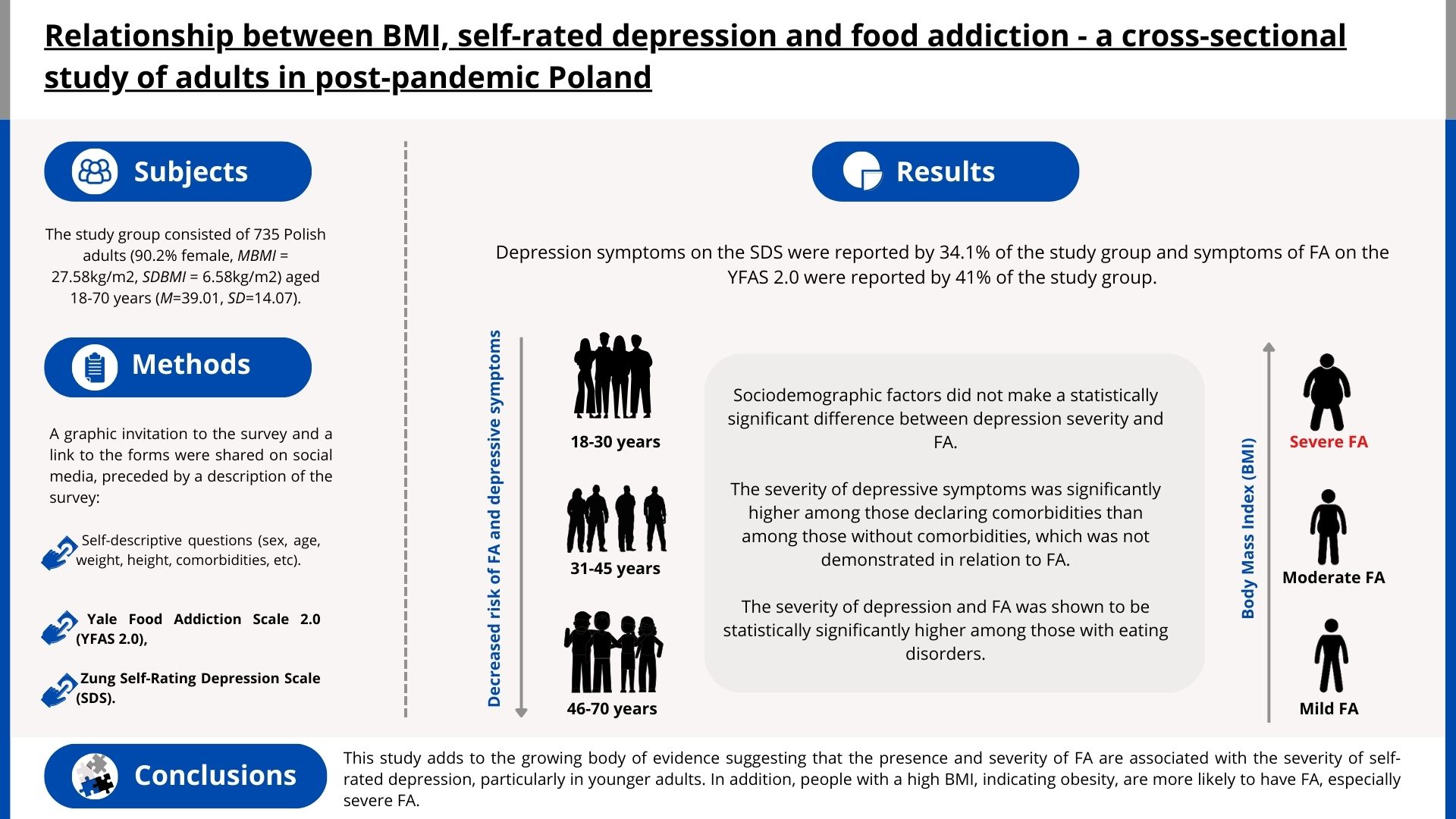

Supplement: Supporting Information — A graphic summary of the conducted research, briefly describing the subjects, methods, most important results, and conclusions. [file 5563257.f1.jpg]
